# Role of Gut Microbiota, Immune Imbalance, and Allostatic Load in the Occurrence and Development of Diabetic Kidney Disease

**DOI:** 10.1155/2023/8871677

**Published:** 2023-12-06

**Authors:** Yi Zhen Han, Hui Juan Zheng, Bo Xuan Du, Yi Zhang, Xing Yu Zhu, Jing Li, Yao Xian Wang, Wei Jing Liu

**Affiliations:** ^1^Dongzhimen Hospital, Beijing University of Chinese Medicine, Beijing, China; ^2^Dongfang Hospital, Beijing University of Chinese Medicine, Beijing, China; ^3^Graduate School, Guangxi University of Chinese Medicine, Nanning, Guangxi, China; ^4^Beijing University of Chinese Medicine, Beijing, China

## Abstract

Diabetic kidney disease (DKD) is a prevailing complication arising from diabetes mellitus. Unfortunately, there are no trustworthy and efficacious treatment modalities currently available. In recent times, compelling evidence has emerged regarding the intricate correlation between the kidney and the gut microbiota, which is considered the largest immune organ within the human physique. Various investigations have demonstrated that the perturbation of the gut microbiota and its associated metabolites potentially underlie the etiology and progression of DKD. This phenomenon may transpire through perturbation of both the innate and the adaptive immunity, leading to a burdensome allostatic load on the body and ultimately culminating in the development of DKD. Within this literature review, we aim to delve into the intricate interplay between the gut microbiota, its metabolites, and the immune system in the context of DKD. Furthermore, we strive to explore and elucidate potential chemical interventions that could hold promise for the treatment of DKD, thereby offering invaluable insights and directions for future research endeavors.

## 1. Introduction

Diabetic kidney disease (DKD) is one of the prevalent microvascular complications associated with diabetes mellitus (DM). A recent investigation of the epidemiology revealed that DKD is the primary cause of chronic kidney disease (CKD) in the United States [1]. Effective treatment methods to delay its progression are still lacking. Specifically, patients with early DKD exhibit mild clinical symptoms and only present with microalbuminuria, which often goes unnoticed by patients [2]. In recent years, there have been advancements in treatment options for DKD patients, such as sodium-glucose transporter 2 (SGLT2) inhibitors [3, 4]. Despite these advancements, some studies have shown that SGLT2 inhibitors are associated with an increased risk of genital infections [5] and are less effective in patients with severe renal impairment [6]. Therefore, it remains crucial to further investigate the pathogenesis and treatment of DKD. Researchers have discovered that patients with end-stage DKD often exhibit significant disruptions in their gut microbiota, immune imbalances, and allostatic loads, with the severity of gut microbiota imbalance being closely tied to the degree of renal injury. Consequently, understanding how to improve DKD by manipulating the gut microbiota to improve the allostatic load caused by immune imbalances has become a focal point of research [7]. The gut harbors a highly intricate microbial ecosystem [8]. Alterations in the composition of gut microbiota and its metabolites can profoundly impact the human immune and metabolic systems [9]. Studies have highlighted the strong connection between gut-microbial ecology and kidney function, particularly in terms of substance metabolism, immune inflammation, gut mucosa, and the composition of gut bacteria [10].

In DKD, the changes in gut microbiota can impact the immunity, ultimately resulting in the acceleration of toxic byproducts. It facilitates the transportation of bacteria and their byproducts into the systemic circulation, causing damage to tissues and organs [11]. The dysbiosis of gut microbiota and gut endotoxins exacerbate kidney injury and proteinuria in DKD patients [12–15]. Short-chain fatty acids (SCFAs) are linked to immune, oxidative stress, and inflammatory responses in DKD [16]. In DKD, dysregulated glucose and lipid metabolism, heightened immune-inflammatory response, medication, diet, and other factors further worsen the dysbiosis of gut microbiota, thereby impacting renal injury [17–21].

Considering these findings, we aim to present the article status on the connection with gut microbiota and immune dysfunction in DKD, elucidating how gut microbiota imbalance contributes to immune dysfunction in DKD mechanism and discussing relevant drug studies.

## 2. The Relationship between DKD and Gut Microbiota and the Immune System

### 2.1. Gut Microbiota and the Immune System

In healthy individuals, the gut harbors over 1,000 diverse types of microorganisms during the neonatal phase. Primarily consisting of bacteria [22], these microbial communities undergo substantial transformations in the weeks following birth [23]. Gut microbiota in infants, thereafter, is influenced by various factors like feeding habits and medications. It is worth noting that after reaching the age of 12, these microbial populations tend to stabilize progressively [24, 25].

Bacteroides, SFB (segmented filamentous bacteria), Bifidobacterium, Lactobacillus, and Bacillus proteus [26] contribute significantly to the immune system and enhancement of the physical barrier in the gut microbiota. Gut monocyte-macrophages are widespread, as they regulate immune cells in the gut and initiate nonspecific immune responses. They not only establish local immunity but also provide resistance against systemic infections [27]. Certain bacterial species, such as Clostridium, have the capacity to stimulate regulatory T cells (Tregs) in the colon and are good to the maturation of the mucosa, as well as shaping the natural killer T (NKT) cells and lymphoid structures [28]. Gut microbiota plays a crucial role in immune responses. The innate immune system involves dendritic cells (DCs), macrophages, granulocytes, and NKT cells. Of these, macrophages and DCs mainly mediate T cell-induced adaptive immune responses, particularly involving T and B cells [29–32].

### 2.2. Relationship between Gut Microbiota and Its Metabolite Imbalance and DKD Immune Imbalance and Allostatic Load

In the process of DKD development, the body undergoes an elevation in its allostatic load, predominantly characterized by persistent inflammation, oxidative stress, and disturbances in glucose and lipid metabolism [33]. This occurrence arises from the unbalance gut microbiota resulting in allostatic load and exacerbates the progression of DKD [34, 35].

In patients with DKD, there is a coexistence of the systemic inflammatory state and impairment of innate immune function. DKD inflammation is induced by macrophages, Toll-like receptors, NLRP3, and nuclear factor-kappa B (NF-*κ*B) [36, 37]. Furthermore, the progression of proteinuria in DKD is promoted by an increase in T or B cells [38]. The escalation of proinflammatory factors worsens the systemic inflammatory state and facilitates the progression of DKD. Simultaneously, the compromised immune system diminishes the body's defense capability and increases susceptibility to infection [39]. Infection ranks second as the leading cause of death in end-stage renal disease [40].

Scientific research has indicated that there is a significant reduction in probiotics that can supply energy for intestinal epithelial cells and secrete SCFAs, thus improving glucose metabolism [41–43]. Among these probiotics, Bacillus proteus has demonstrated the ability to enhance the host's defense mechanisms by inducing the biosynthesis of lipopolysaccharide (LPS) and maintaining LPS levels for immune homeostasis [44, 45]. For example, exposing germ-free mice to LPS improves abnormalities in the colonic mucosa [46]. When LPS is recognized, Toll-like receptor 4 (TLR4) activates MyD88, which promotes pathogen clearance and preserves the health of intestinal epithelial cells [47]. Mice without MyD88 have demonstrated increased susceptibility to gut damage [48]. In patients with DKD, there is a positive correlation between Lactobacillus reuteri and the urinary albumin-to-creatinine ratio [49]. Additionally, the composition of the gut microbiota in DKD patients exhibits alterations in the proportion of dominant flora and their metabolites under different conditions [50]. Furthermore, in patients with early-stage DKD, an increase in Proteobacteria has been observed, causing the activation of macrophages, exacerbating the chronic low-grade inflammatory state [51]. In patients with end-stage DKD, an imbalance in the ratio of Firmicutes and Bacteroides and a low level in SCFA production have been noted, potentially disrupting glucose metabolism [52, 53].

## 3. The Role of Gut Microbiota in the Development of Diabetic Nephropathy by Affecting the Innate Immune System

### 3.1. Composition of the Gut Microbiota in the Innate Immune System

The innate immune system protects the body, and intestinal epithelial cells play a key part by secreting mucins and antimicrobial proteins (AMPs). These cells work alongside gut neutrophils to establish a physical barrier, preventing the entry of harmful pathogens [54–57]. In a healthy colon, most of the resident cells receive supplementation from monocytes through a mechanism dependent on C-C chemokine receptor type 2. This mechanism is characterized by tolerance and a lack of response to TLR stimulation. However, when there is an imbalance in gut microbiota, the normal process of monocyte-macrophage maturation is disrupted. As a result, macrophages produce tumor necrosis factor-alpha (TNF-*α*) and interleukin-6 (IL-6) when exposed to TLR stimulation, ultimately leading to kidney inflammation [58]. Peptidoglycan is the component of the innate immune system and found in the bacterial cell walls. Peptidoglycan facilitates Streptococcus pneumoniae and Staphylococcus aureus and initiates the immune response [59]. Additionally, butyrate, a type of SCFAs, has the chance to decrease NF-*κ*B signal transduction in DCs and neutrophils. Bile acids (BAs) also contribute to immune regulation by protecting the physical barriers and reducing inflammation through their effects on cytokine bioactivity.

### 3.2. Dysregulation of Gut Microbiota and Its Metabolites Affects Both Innate Immunity and the Role of Persistent Inflammation in the Pathogenesis of DKD

DCs and macrophages respond to stimulation with TLR ligands [60–63] and produce interleukin-10 (IL-10) under normal conditions [62, 63]. In the context of DKD, these immune cells attract and activate humoral immunity through TLR, initiating a rapid inflammatory response [64] that ultimately leads to kidney damage. Additionally, the integrity of the gut barrier becomes compromised, leading to the accumulation of toxins. These toxins activate immune system, which lead to persistent systemic inflammation. Hence, persistent inflammation caused by intestinal barrier damage can aggravate DKD.

Research has indicated that DCs and macrophages are capable of modulating the innate immune system by exerting TLR-mediated control [65]. In a study involving patients with microalbuminuria, glomerular TLR4 expression was significantly higher in the glomeruli and tubules [66]. Another study utilizing an animal model demonstrated that enhanced TLR2 expression in renal tubules and macrophages led to a proinflammatory environment and the occurrence of microalbuminuria [67, 68].

Patients with DKD exhibit changes in the composition of their gut microbiota, such as an increase in the abundance of Escherichia-Shigella bacteria that can breach the intestinal barrier [69]. This breach allows the gut microbiota to move into other areas of the body, triggering the activation of innate immune cells and the upregulation of TLR2 and TLR4-related pathways in the kidney. These immune responses result in the production of proinflammatory cytokines, leading to immune dysfunction, heightened infection susceptibility, and kidney damage specifically in DKD patients [67, 70–72]. Interestingly, nonpathogenic Salmonella bacteria have been found to counteract renal inflammation by inhibiting the NF-*κ*B signaling pathway [73, 74]. Several studies have demonstrated that dysregulation of metabolic endotoxin/lipopolysaccharide levels in a rat model of DKD leads to activation of the NF-*κ*B signaling pathway, increased levels of inflammatory cytokines in both the bloodstream and kidney, and activation of the innate immune system [75]. However, experiments using TLR4 gene deficiencies in vivo have shown that TLR4 receptors have the ability to detect and activate harmful and endogenous damage signals, subsequently causing kidney injury and fibrosis in DKD patients [36, 76–78].

Furthermore, the presence of mitochondrial antiviral signaling protein (MAVS) is crucial for maintaining gut integrity and innate immunity. In one study, the gut integrity of a DKD mouse model was impaired by MAVS gene knockout, resulting in the detection of gut-derived Klebsiella oxytoca, interleukin-17 (IL-17), and kidney injury molecule-1 (KIM-1) in the circulation and kidney. The inhibition of MAVS led to gut epithelial cell inflammation and consequent renal injury [79].

### 3.3. Dysregulation of Gut Microbiota and Its Metabolites Affects Both Innate Immunity and the Role of Glucose and Lipid Metabolism in the Pathogenesis of DKD

In DKD, the gut microbiome is disrupted, and the balance between good and bad bacteria is altered. This imbalance then results in the disruption of carbohydrate and lipid metabolism in the body [12, 17].

The first aspect is primarily composed of the presence of advanced glycation end products and their stimulation of the expression of transforming growth factor *β*. This leads to increased glycation of proteins, which causes damage to renal tubular epithelial cells and local kidney damage [10]. On the other hand, the second aspect is mainly seen through the occurrence of lipid peroxidation, inhibition of extracellular matrix degradation, infiltration of monocyte-macrophages in the kidney, and the induction of glomerulosclerosis and tubulointerstitial damage.

Increased carbonylation and glycation of proteins, one of the main manifestations of allostatic load [80], disrupts the structure and function of various proteins in DKD, leading to cell dysfunction and organ damage. Proinflammatory cytokines are glycosylated proteins with immunomodulatory functions [36].

Research has shown that TLRs and NLRP3 inflammasomes can produce proinflammatory factors to mediate sterile tubulointerstitial inflammatory response. The bradykinin-releasing enzyme-bradykinin system can activate bradykinin, and its receptors can lead to renal injury. The kidneys were protected, and proteinuria was reduced upon treatment with SCFAs. This effect is related with the ability of SCFAs to inhibit glycosylated proteins [81]. Furthermore, the enhanced expression of TLR2 contributes to the upregulation of chemokine MCP1/CCL2 in both glomeruli and tubular epithelium, potentially leading to impaired renal function in individuals with diabetes [82].

### 3.4. Dysregulation of Gut Microbiota and Its Metabolites Affects Both Innate Immunity and the Role of Oxidative Stress in the Pathogenesis of DKD

The occurrence of DKD is related to an asymmetry in the gut microbiota and disruptions in lipid metabolism, resulting in decreased connective protein expression [83]. Consequently, it causes heightened gut permeability and translocation, ultimately impacting DKD oxidative stress pathway via the innate immune system, thereby contributing to disease progression.

As proficient stimulators of neutrophils, SCFAs elicit the proliferation and migratory response of neutrophils, generating reactive oxygen species (ROS) to safeguard beneficial bacteria. Concurrently, SCFAs impede NF-*κ*B and foster the expansion of T cells [84, 85]. Moreover, SCFAs have the ability to fortify the physical barrier via intestinal epithelial cells, thus thwarting gut microbiota leakage [83]. Absence of GPR43 in colitis mice led to reduced colonic neutrophil counts, in contrast to the wild-type colitis mice [86].

**Figure 1:**
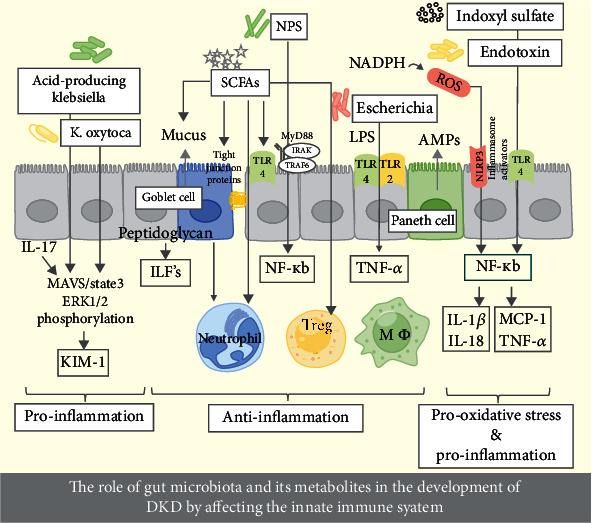
The role of gut microbiota and its metabolites in the development of DKD by affecting the innate immune system.

Toxic metabolites build up in the systemic circulation and trigger ROS through the NADPH pathway. This leads to the activation of the NF-*κ*B and causes inflammation, resulting in proteinuria and damage to podocytes [87]. One specific factor contributing to oxidative stress is the NLRP3 inflammasome complex, which is driven by ROS in mitochondria and mitochondrial dysfunction. The activation of the NLRP3 inflammasome complex can stimulate interleukin-18 (IL-18), thereby exacerbating kidney injury in DKD [88, 89]. Inflammasome activation has been observed in podocytes and endothelial cells, in the context of hyperglycemia, obesity, and lipotoxicity [90–92] (Figure 1).

## 4. The Role of Gut Microbiota and Its Metabolites in the Development of Diabetic Nephropathy by Affecting the Adaptive Immune System

### 4.1. Composition of the Gut Microbiota in the Adaptive Immune System

The arrangement of gut microbiota components within the adaptive immune system primarily consists of B cells, T cells, and a majority of metabolites. The primary functions attributed to B cells involve their interaction with gut antigens [93] and the prevention of gut antigens from entering the bloodstream [94]. The equilibrium of gut immunity heavily relies on CD4+ T cells that primarily reside in the gut LP. These T cells can be classified as effector or helper CD4+ T cells, among which helper T cells can be further categorized into T-helper 1 (Th1), T-helper 17 (Th17) cells, and so forth [26]. Tregs can produce anti-inflammatory cytokines like IL-10, which aids in reducing MHC-II expression in monocyte-macrophages. Additionally, Tregs can directly impede the proliferation of proinflammatory factors and suppress the production of chemokines in DCs in an autocrine manner [95].

SCFAs inhibit inflammation through their interaction with free fatty acid receptors and GPR complex signal transduction [96–99]. Furthermore, the gut microbiota, including Lactobacillus, Clostridium, Bifidobacterium, and Enterococcus, plays a role in controlling bile acid synthesis [100] and regulating adaptive immune suppression and inflammation [101, 102]. By decomposing into indole and its derivatives, tryptophan helps maintain gut immune homeostasis by regulating adaptive immunity. The accumulation of a uremic toxin, trimethylamine n-oxide (TMAO), in the circulation is linked to renal injury [103]. Additionally, polyamines are essential in maintaining the epithelial barrier, promoting intestinal epithelial cell persistence, and inhibiting inflammation. Interestingly, the levels of phenyl sulfate (PS), a derivative of L-tyrosine in the gut, linked to the development of proteinuria in DKD.

### 4.2. Gut Microbiota and Its Metabolites Affect Both Adaptive Immunity and the Role of Persistent Inflammatory Response in Diabetic Nephropathy

Persistent inflammation in DKD is closely related to an imbalance in Th/Treg cells. Research indicates that the inflow rate of Tregs is associated with proteinuria, as well as glomerular and tubular damage, ultimately leading to fibrosis [104]. The gut microbiota, along with its metabolites, can directly impact adaptive immunity's T and B cells while indirectly influencing other immune cells, thereby contributing to the generation of persistent inflammatory responses [104].

The gut's Bacteroides fragilis plays a role in protecting the kidneys through its systemic induction of Th1 responses [105, 106]. DKD patients show increased concentration of interferon-g (IFN-g), which is linked to the production of interferon-*γ* (IFN-*γ*) and Th2, ultimately leading to the development of DKD [107–109]. Additionally, Bacteroides fragilis can enhance its colonization ability and increase IL-10 secretion by increasing the induction of FOXP3+ Tregs through octa capsular polysaccharide (PSA) [110–112]. This process helps regulate the biased Th/Treg levels.

Polysaccharide A of Bacteroides, which is known for its T cell-dependent properties and maintaining the gut barrier, was reduced in the population with CKD [113]. PSA restores the balance of Th1 and Th2 and inhibits renal inflammation by stimulating regulatory T cells to secrete IL-10. Additionally, Bacteroides play a role in the BAs and SCFAs, which have been shown to slow the advancement of DKD [114–116]. BAs, such as tauroursodeoxycholic acid (TUDCA), exert their effects on the immunity by increasing the bile acid receptor Gpbar1 (TGR5) and farnesoid X receptor (FXR), thereby delaying the progression of DKD [117].

Promoting Th17 cells and enhancing the defense ability of the intestinal mucosal surface, SFB has the potential to reduce infection risk and safeguard kidney health [118]. The gut serves as the entry point for Tregs, which activate to maintain a balance in effector cell activity by suppressing inflammation and promoting immune tolerance [119, 120]. Research suggests that DKD patients exhibit decreased proportions of Tregs in their peripheral blood. Furthermore, the transfer of Tregs in diabetic mice has been found to effectively ameliorate DKD [121]. Moreover, evidence indicates that the colonization of mice by SFB triggers Th cell [122]. Specifically, T cells equipped with SFB-specific antigen receptors differentiate into Th17 cells, which are responsible for the production of cytokines [123, 124].

In the gut lumen, B cells have the capability to secrete IgA (sIgA) [82, 125]. Alongside, the secretion of IgA is also facilitated by TGF-*β* and IL-10 [93]. The primary function of sIgA is to create a protective barrier for epithelial cells against pathogens and aid in maintaining gut immune homeostasis [94]. sIgA can control systemic adaptive T cell responses through immune rejection and maintain gut immune homeostasis against commensal bacteria [126, 127].

Gut immune responses are facilitated by gut macrophages [64, 127]. Recent research has demonstrated that mice with F4/80 gene knockout exhibit an inability to develop tolerance or reduce antigen-specific CD8+ Tregs and gut macrophages upon ingestion of soluble antigens [128]. Neutrophils play a role in decomposing bacteria and cellular fragments in the gut [129]. Gut DCs transport antigens from gut bacteria to the mesenteric lymph nodes (MLN) [130–132]. These DCs are generally less responsive under normal conditions, which helps maintain gut immune tolerance and diminish inflammation. They accomplish this by promoting the development of T cell that tolerate commensal bacteria and food antigens [60, 133]. Similarly, gut macrophages, like DCs, express low CD86, CD80, and CD40 levels [61, 134, 135]. Additionally, they increase IL-10, thereby reducing inflammation [62, 63].

Uremic toxins have a significant role in the progression of CKD [136]. The inhibition of HDAC activity by SCFAs has implications for different immune system cells [96]. Specifically, it can promote the differentiation of T cells into effector and contribute to the inflammatory response of DKD while also providing protection to the kidneys [137]. Tryptophan, another metabolite produced by gut microbiota, has various effects on gut immune cells and epithelial cells [138, 139]. It can produce inflammatory factors and hinder the development of Th17 cells [140]. The conversion of tryptophan precursors into ligands for the aryl hydrocarbon receptor (AHR) [141, 142] is facilitated by lactic acid bacteria and bifidobacteria [142]. These ligands then bind to the AHR, leading to the translocation of the receptor into the nucleus. Through this mechanism, the AHR regulates IL-22 and IL-17, resulting in functional changes and pathological alterations in diabetic mice [143–145]. Moreover, the activation of innate lymphoid cells (ILCs) and T cells, along with the production of IL-17 and IL-22, has been observed [138]. Of note is IL-22 against the colonization of pathogenic microbiota [146]. Research has shown that mice deficient in AHR or AHR ligands exhibit alterations in the composition of gut microbiota. AHR-deficient mice have lower levels of ILCs and IL-22 [147, 148]. These research indicate a relationship between AHR activity and gut microbiota metabolism in DKD [146, 149, 150]. Another investigation discovered that the production of compounds derived from indole by Lactobacillus reuteri might stimulate the conversion of CD4+ T cells within the gut epithelium into CD4+ CD8+ double-positive intraepithelial lymphocytes. This transformation is beneficial for maintaining the immune balance within the intestines [151]. The presence of polyamines can modify T cells by rising the expression of connexin and promoting the secretion of mucus, thus regulating the levels of inflammatory substances. Following the administration of PS, the foot processes vanished, podocytes suffered damage, the glomerular basement membrane thickened, and there was a rise in the mRNA levels of monocyte chemoattractant protein-1 (MCP-1) and fibronectin 1 (Fn1) in kidney tissue. In diabetic patients, the levels of inflammation and renal fibrosis were exacerbated [152].

### 4.3. Gut Microbiota and Its Metabolites Affect Both Adaptive Immunity and the Role of Glucose and Lipid Metabolism in the Pathogenesis of Diabetic Nephropathy

In hyperglycemia and hypercholesterolemia, DKD leads to an imbalance of gut microbiota, and it will further activate the adaptive immune system, resulting in renal inflammation [18]. Simultaneously, excessive lipid accumulation reduces SCFAs, inhibiting insulin sensitivity and causing renal injury [19].

Increased carbonylation and glycation of proteins, such as thrombin in the body, can activate receptors by activating proteases in kidney cells and recognize glycated proteins and/or glycated complement regulatory protein dysfunction through mannan-binding lectins, lead to activation of complement cascade [36], and promote renal inflammation and renal fibrosis in DKD. Meanwhile, the dysfunction of glycated albumin can cause renal injury and fibrosis by upregulating TGF-*β* [153].

### 4.4. Gut Microbiota and Its Metabolites Affect Both Adaptive Immunity and the Role of Oxidative Stress in the Pathogenesis of Diabetic Nephropathy

**Figure 2:**
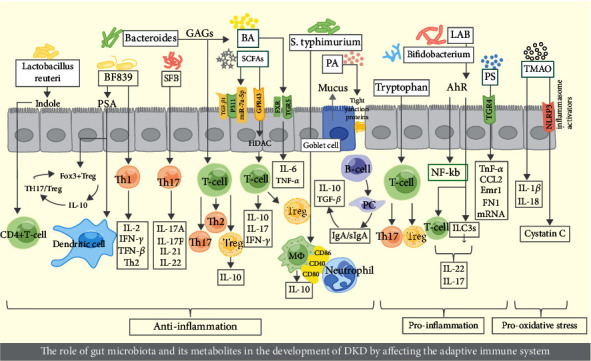
The role of gut microbiota and its metabolites in the development of DKD by affecting the adaptive immune system.

When renal function declines, toxic levels of TMAO [154–156] accumulate in the circulation due to the stimulation of glomerular and tubular damage by uremic toxins, promoting oxidative stress [103]. Mice were fed with TMAO or its precursor choline, resulting in renal tubular damage and renal fibrosis induction [157]. Additionally, TMAO has been shown to aggravate renal fibrosis by impacting the secretion of IL-1*β* and IL-18 through the inflammasome channel NLRP3 [15]. Sun et al. also discovered that 3,3-dimethyl-1-butanol prevented high-fat diet-induced renal fibrosis [158] (Figure 2).

## 5. The Role of Gut Microecological Interventions in Retarding the Progression of DKD

**Figure 3:**
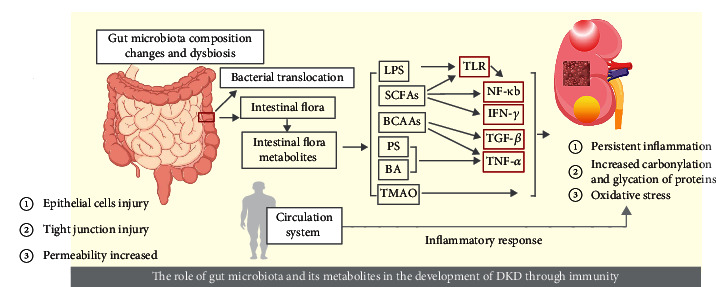
The role of gut microbiota and its metabolites in the development of DKD through immunity.

DKD is impacted by disturbances in gut microbiota, which result in elevated levels of metabolites linked to gut microbial activity and compromised integrity of the gut barrier. Consequently, a sustained state of systemic inflammation arises, accompanied by weakened immune function. Hence, potential treatments for DKD are modulation of gut microbiota as well as improvement of the immune system (Figure 3).

### 5.1. Dietary Interventions

Dietary interventions are the most effective influences among the exogenous factors affecting gut microbiota [159, 160]. According to existing studies, it is evident that the progress and onset of DKD can be altered through sensible dietary interventions due to the strong correlation between diabetes development and nutritional habits [161].

#### 5.1.1. Dietary Fiber (DF)

DF primarily consists of nonstarch polysaccharides [162]. The inclusion of dietary fiber decelerates the renal dysfunctions through the release of SCFAs and the reduction of inflammation in DKD. Previous research [12] demonstrated that when resistant starch and high fiber diets supplemented with guar gum and cellulose were administered to diabetic mice, there was an expansion observed in SCFA-producing Prevotella and Bifidobacterium genera, resulting in elevated SCFA levels and a decrease in pathological Bifidobacterium and CD68+ cells. Moreover, mice in the RS group exhibited a decrease in urinary albumin/creatinine, albuminuria, and mRNA expression of inflammatory cytokines and fibrosis-related genes. Dietary fiber has a protective and ameliorative role in DKD by modulating gut microbiota's production of SCFAs and regulating crucial pathways associated with innate immunity, inflammation, and macrophage recruitment.

#### 5.1.2. High Linoleic Acid (LA) Diet

LA is a typical polyunsaturated fatty acid (PUFA) in plant-derived oils, mainly derived from vegetable oils and nuts in the diet [163]. A diet high in linoleic acid is typical of the Mediterranean diet, and previous studies have suggested that in patients with DKD, adopting the Mediterranean diet could potentially present a lifestyle approach that effectively postpones kidney deterioration [164, 165]. A study conducted on 366 patients as part of a clinical trial revealed that an enhanced intake of polyunsaturated fatty acids had a notable association with a decreased preponderance of DKD [166]. Animal experiments also supported these findings, wherein rats induced with T2DM through STZ and NA were administered a polyunsaturated fatty acid-rich diet. The diet effectively reversed the rise in the ratio of Bacteroides to Firmicutes within gut tract and also decreased IL-6, IL-1*β*, TNF-*α*, and IL-17A [167]. This suggests that a diet rich in linoleic acid can potentially regulate intestinal flora, leading to a delay in the progression of DKD and proposing it as a significant dietary intervention for DKD patients.

#### 5.1.3. Cereals

Cereals, which are extensively cultivated food crops across various regions globally, have demonstrated to alleviate inflammatory components [168]. It has been highlighted in a research investigation that cereals could potentially safeguard against high-intensity inflammation and morphological deviations in DKD [169]. This protection mechanism is achieved through the facilitation of SCFA release and the restoration of gut microbiota, consequently restraining the excessive expression of MCP-1 and TNF-*α*.

#### 5.1.4. Astaxanthin (AST)

AST is a naturally occurring ketocarotenoid present in many microalgae. It exhibited numerous activities both in laboratory settings and in living organisms [170]. Studies have indicated that when mice with diabetes were exposed to a high-fat diet, either with 01.0% (AST) or 02.12% (AST) for a period of one week, the addition of AST effectively delayed the progression of kidney damage by inhibiting LPS, TMAO, and IL-1 and by modulating the NF-*κ*B signaling pathway, in comparison to the DKD group. Furthermore, including AST as part of the diet leads to gut microbiota composition changes. This is supported by the decrease in bacteria like Coriobacteriaceae UCG-002 and the increase in probiotics Ruminococcaceae [171]. AST supplements might lead to ameliorate kidney injury associated with DKD by promoting a healthier intestinal flora and subsequently influencing immune factors.

### 5.2. Probiotics, Prebiotics, and Synbiotics (PPS)

Synbiotics include probiotics, live microorganisms beneficial to the host in specific quantities, including Lactobacillus and Bifidobacterium, and prebiotics, substrates selectively used by host microorganisms [172, 173].

Studies have shown that synbiotics slow the progression of kidney damage in DKD patients by correcting gut microbiota balance, modulating the host immune response, reducing proinflammatory factors, and improving inflammation [10, 174]. Dai et al. [175] showed that moutan cortex polysaccharide ameliorates (MC-Pa) increased Lactobacillus, increasing SCFAs and improving serum IL-6. It alleviated the structural abnormalities of tubules in DKD rats.

### 5.3. Metabolic Regulation

#### 5.3.1. SCFAs

The effect of SCFAs on DKD has been studied, and it has been found that SCFAs can help regulate inflammation in DKD [176]. Researchers have shown that SCFA supplementation can alleviate renal inflammation in DKD by acting on the immune response process [177]. In particular, butyrate, a type of SCFA, has been found to have immunomodulatory effects [37]. It can also modulate the intestinal barrier and regulate insulin sensitivity. In a study, exogenous butyrate was found to significantly reduce NF-*κ*B and MCP-1 and IL-1*β*, which are markers of inflammation, through a GPR43-*β*-arrestin-2 mechanism. This study also showed that butyrate restored inflammatory injury in the kidneys [178–180]. Another study applied sodium-butyrate to DKD mice and indicated that it reduced TGF-*β*, Fn, IL-6, and MCP-1, thus improving DKD by inhibiting renal inflammation [181, 182]. Furthermore, research has shown that butyrate supplementation can decrease kidney fibrosis by mediating the TGF-*β*1 pathway [183]. Overall, these findings suggest that exogenous SCFAs, particularly butyrate, have the potential to prevent and treat DKD by inhibiting renal inflammation and fibrosis gene expression.

#### 5.3.2. BAs

Gut microbiota in the gut convert primary BAs into secondary BAs [184, 185]. This conversion process was demonstrated in a study, where tauroursodeoxycholic acid (TUDCA) had a beneficial effect on db/db and STZ-induced DKD mice. Specifically, TUDCA attenuated the expression of IL-6, TNF-*α*, and collagen 1 *α* 2. The mechanism of action involved targeting FXR or TGR5 pathways, leading to the improvement of glomerular and tubular injury [13]. Importantly, TGR5 has been found to decrease renal inflammation by inhibiting the NF-*κ*B signaling [117]. This suggests that exogenous BAs could be therapeutic agents in DKD.

#### 5.3.3. Branched-Chain Amino Acids (BCAAs)

BCAAs are composed of leucine, valine, and isoleucine [186]. It has been demonstrated that BCAAs possess the ability to hinder the expression of TGF-*β* in order to mitigate the damage caused by diabetic kidney injury [14]. A study recently found that administering high quantities of BCAAs prevented kidney weight in mice with DKD, additionally, moderate amounts of BCAAs led to a reduction in TGF-*β* mRNA and mitigated oxidative stress by activating the TGF-*β* pathway, thereby lessening the severity of DKD kidney injury. Furthermore, providing oral supplementation of BCAAs to individuals may also enhance their appetite and overall nutritional status [187].

#### 5.3.4. TMAO

TMAO is a group of metabolites that form when trimethylamine (TMA) is oxidized through the metabolism of gut microbiota [188]. According to research, TMAO promotes inflammation in the kidneys, ultimately leading to interstitial dysfunction [189]. A study found that the intake of TMAO at 0.2% (*w*/*v*) resulted in higher kidney index and urinary protein in DKD rats induced by 35 mg/kg STZ compared to the DKD group without TMAO intake. Furthermore, the TMAO group also exhibited a significant increment IL-18 and IL-1*β*, which further intensified kidney inflammation [15]. Therefore, it can be inferred that elevated levels of TMAO may exacerbate DKD.

### 5.4. Fecal Microbial Transplantation (FMT)

FMT alters the gut microbial composition of patients with DKD by applying fecal solutions from healthy donors [190]. It has been shown [191] that FMT can slow down the process of kidney injury in DKD by rebuilding abnormal gut microbial ecology. It was shown that FMT leads to increase in Odoribacter spp. In the black and tan brachyury ob/ob mouse model of DKD, there is a decrease in urine albumin-creatinine ratio (UACR) and TNF-*α* and an improvement in the tendency of insulin resistance [192]. Cai et al. used resveratrol-treated db/db mice as FMT donors. They showed a reduction in the proportion of thick-walled Enterobacteria and Ferribacterium as well as an increase Proteobacteria and abundance of Odoribacter spp. and Bacteroides, as well as urinary albumin excretion rates (UAER), serum creatinine, and kidney inflammatory factor level reduction, suggesting that the reduction of inflammation is a key mechanism by which FMT protects renal function in DKD [193].

### 5.5. Natural Drugs

Extensive research findings indicate that the utilization of natural medications exhibits a positive impact on the management of DKD by restraining inflammation and oxidative stress. In addition, these drugs help modulate the gut microbiota and related metabolic processes [194, 195].

#### 5.5.1. Chinese Herbal Monomers and Their Extracts

*(1) Bupleurum Polysaccharides (BP)*. Bupleurum, a perennial herb from Umbelliferae, yields a polysaccharide known as BP. According to studies, BPs may act by interrupting high mobility group box-1 protein-TLR4 [196]. This disease suppresses renal inflammation and fibrosis processes [197]. In STZ-induced DKD mice, BP has the potential to reverse the reduction in Bacteroidetes abundance and the increase in Proteobacteria and Ferribacterium abundance. In addition, studies indicate that BP can lower TLR1 levels. In doing so, it reduces the inflammatory response, repairs the intestinal barrier to reduce LPS content, and ultimately addresses glomerular hypertrophy and glomerular hyperplasia. Moreover, BP also shows promise in reducing urinary albumin in diabetic mice [198]. Given these findings, BP holds the potential to enhance DKD by modulating the gut microbiota in the kidney.

*(2) Cordyceps cicadae Polysaccharides (CCP)*. CCP, the primary active compound found in the parasitic medicinal fungus Cordyceps sinensis, has garnered attention for its therapeutic potential in the treatment of diabetes [199]. Multiple studies have underscored CCP treatment's ability to demonstrate hypoglycemic properties, thereby reducing tissue damage typically associated with diabetes. Furthermore, CCP administration has exhibited notable changes in the ratio of Firmicutes/Bacteroidetes, including increased copiousness of Odoribacter, Bacteroides, Alloprevotella, Mucispirillum, and Parabacteroides while the populations of Lactobacillus and Helicobacter have shown significant decrease [200]. In rats with streptozotocin-induced DKD, CCP supplementation has proven effective in mitigating renal fibrosis by upgrading the maturation of Bacteroidetes and Lactobacillus communities while reducing the presence of LPS-producing bacteria. These effects are attributed to CCP's regulatory impact on gut microbiota dysbiosis by inhibiting TLR4. In turn, this regulation leads to lowered serum engrossment of TNF-*α*, resulting in significant improvements in 24-hour urine volume and Scr levels. The positive impact of CCP on renal inflammation in DKD rats is further evidenced by the amelioration of collagen fiber accumulation in the glomerular mesangial area, lipid accumulation in kidney tissue samples, and the thickening and widening of the kidney tubular basement membrane [35].

*(3) Cornus*. Cornus, a deciduous perennial tree or shrub, exhibits antifibrotic effects in DKD by inhibiting TGF-*β* and hypoxia-inducible factor-1 (HIF-1) signaling pathways, as determined through network pharmacological analysis [201, 202]. The efficacy of Cornus in reducing glomeruli nodular sclerosis and kidney interstitial edema in STZ-induced DKD rats, along with its ability to decrease TGF-*β* and enhance abundance of gut lactobacilli to elevate SCFA content, suggests that it can be used to treat DKD by restoring gut microbiota's abundance, increasing SCFA levels, and diminishing inflammatory infiltration [203].

*(4) Ginsenoside Compound K (CK)*. Ginsenoside, the primary extract derived from ginseng, which is a perennial plant belonging to the Wujia family, is a steroidal compound [204]. One of the major metabolites of ginseng is known as ginsenoside compound K. Research conducted in the past has illustrated the favorable impacts of CK on DKD, effectively alleviating glomerular injuries [205]. Moreover, it has been found that CK supplementation of a diet containing 0.03% dosage significantly reduces proteinuria, glomerular dilatation, glomerulosclerosis, and inflammatory infiltration. This reduction is achieved by diminishing the level of Bacteroides and increasing Lactobacillus levels. Furthermore, TGF-*β*1 expression in the kidney is reversed, effectively inhibiting NF-*κ*B and subsequently decreasing IL-6 and IL-1*β* levels. Lastly, decreased serum imidazole propionate (IMP) will guide the downregulation of protein expression induced by IMP [206].

*(5) Magnesium Lithospermate B (MLB)*. MLB is a constituent found in water extracts of Salvia miltiorrhiza, a perennial herb belonging to the Sage genus in the Labiatae family [207]. Experimental investigations indicate that MLB can mitigate kidney injury caused by STZ-induced DKD in mice by inflecting the composition of gut microbiota derived from it [208]. The oral administration of MLB effectively suppressed the unfreezed of inflammatory cells caused by BA and resulted in a decrease in urinary albumin levels over a 24-hour period in rats with STZ-induced DKD, thus slowing down the progression of kidney injury. Moreover, MLB intervention significantly reduced the abundance of Shigella and Aspergillus species, as well as the level of BAs in the feces of the rats [209].

*(6) Resveratrol*. Resveratrol is a class of polyphenolic compounds of distyrene, which are widely found in various Chinese herbs, such as Polygonum cuspidatum and mulberry, and is a natural therapeutic products for the treatment of T2DM [210]. Previous network pharmacological studies have depicted that resveratrol is an efficacious drug in DKD [211]. Research has demonstrated that administering resveratrol orally in mice can rectify the Firmicutes/Bacteroides ratio and diminish levels of inflammatory factors, serum creatinine, blood urea nitrogen, and urinary 24-hour microalbuminuria in db/db mice. [193]

#### 5.5.2. Chinese Herbal Compound

*(1) Qing-Re-Xiao-Zheng Formula (QRXZF)*. QRXZF is a traditional Chinese medicine (TCM) prescription. Gao et al. showed that QRXZF (2 g/ml) reversed the increases in UACR and improved thylakoid matrix expansion and tubulointerstitial injury in STZ-induced DKD mice, which may be related to the fact that QRXZF reversed the increase in Desulfovibrionaceae and Desulfovibrio in DKD mice, reduced gut-derived LPS in the blood, and inhibited inflammatory signaling pathway [212]. In their study, Shen et al. demonstrated the potential of Salvia miltiorrhiza and Astragalus membranaceus in enhancing DKD. The predominant bacteria involved in glycolipid metabolism were identified as Lactobacillus murinus and Akkermansia muciniphila [213].

*(2) Shenyan Kangfu Tablet (SYKFT)*. SYKFT comprises a combination of thirteen Chinese herbal medicines, namely, Panax quinquefolius, Panax ginseng, Rehmannia glutinosa, Eucommia ulmoides, Dioscorea oppositifolia, Salvia miltiorrhiza, Leonurus artemisia, Smilax glabra, Oldenlandia diffusa, Glycine max, Imperata cylindrica, Alisma plantago-aquatica, and Platycodon grandiflorus. Chen et al.'s research indicates that SYKFT led to a reduction in TNF-*α* levels in the kidneys, along with an improvement in gut microbiota. Specifically, SYKFT increased the presence of Firmicutes while decreasing Bacteroidetes, resulting in the alleviation of kidney insufficiency and kidney inflammation in db/db model DKD mice [214]. CK, a component of Panax ginseng, reshaped the microbiota exhibiting potential in combating inflammation associated with DKD [206].

*(3) Tangshen Formula (TSF)*. The TSF was obtained from a combination of 7 different herbs. The ratio of these herbs used in the extraction was 10 : 5 : 4 : 3.4 : 3 : 2 : 1 (*W*/*W*). Previous studies by Zhao et al. have designated that TSF can increase the presence of bifidobacteria, while also stamping the release of intestinal-derived inflammatory substances (IS) and LPS. In addition, TSF was found to suppress the TLR4/c-Jun N-terminal kinase (JNK) and NF-*κ*B signaling pathways in the kidneys. This suppression resulted in a decrease in microalbuminuria and serum creatinine levels, as well as the inhibition of moderate expansion of the kidney thylakoid matrix, luminal dilatation, and tubular interstitium of rats with DKD induced by STZ and uninephrectomy [215]. Furthermore, a purified preparation of anthraquinone-glycosides derived from rhubarb, which are monomeric compounds found in rhubarb, has been shown to reduce inflammation in individuals with DM [216] (Table 1).

## 6. Summary and Prospects

The correlation between DKD and the microbiota residing in the gastrointestinal tract, along with its metabolic byproducts and their interaction with the innate and adaptive immune systems, has progressively been elucidated. This is closely related to the allostatic load and immune imbalance caused by gut microbiota translocation and dysregulation between gut microbiota and DKD. However, current research still needs to clarify the role of certain bacterial strains such as Bacteroidetes, Firmicutes, Fusobacteriota, and Actinobacteriota, especially the role of the abundance of Bacteroides and Firmicutes in DKD inflammation and intestinal barrier permeability. In addition, natural drugs have significant advantages in treating DKD, and existing studies lack the immune mechanism between herbal compounds and gut microbiota, which will become a future research direction for DKD prevention and treatment.

## Figures and Tables

**Table 1:** Experimental study of natural drugs on gut microbiota in DKD.

Ingredient	Source	Moulding method	Effect on gut microbiota	Action mechanism	Related literature
Bupleurum polysaccharides (BP)	Bupleurum	STZ (1 mg/kg)	Reverse the decrease of Bacteroides abundance and the increase of Proteobacteria and Ferribacterium abundance.	Inhibit TLR1 levels, reduce TNF-*α* and IL-6 levels in the kidney, and improve intestinal barrier.	[189]
Cordyceps cicadae polysaccharides (CCP)	Cordyceps sinensis	STZ (0.1 mg/kg)	Increase the abundance of Lactobacillus and Bacteroidetes, decrease Proteobacteria and Deferribacteres.	Block TLR4/NF-*κ*B and TGF-*β*1 signaling pathways, decrease the concentrations of serum TNF-*α*, IL-1*β*, and IL-6.	[35]
Cornus	Cornus	STZ (35 mg/kg)	Increase the abundance of gut lactobacilli and increase SCFA content.	Reduce inflammatory infiltration.	[190]
Ginsenoside compound K	Ginseng	db/db mice	Decrease the level of Bacteroides and increase the level of Lactobacillus.	Reverse the upregulation of TGF-*β*1 expression, inhibit NF-*κ*B, decrease the expression of IL-6 and IL-1*β*, and downregulate the expression of the IMP-induced TLR4 signaling pathway.	[191]
Magnesium lithospermate B (MLB)	Salvia miltiorrhiza	STZ (40 mg/kg)	Reduce the abundance of Shigella and Aspergillus species.	Regulate BA metabolism, restore intestinal barrier integrity, and inhibit inflammatory cell release.	[192]
Resveratrol	Polygonum cuspidatum, mulberry	db/db mice	Restore the proportion of Firmicutes/Bacteroides.	Reduce the kidney mRNA levels of TNF-*α*, IFN-*γ*, IL-6, and IL-1*β*.	[217]

## Additional Points

*Highlight*. The connection between the allostatic load of DKD and gut microecology is robust, involving immunity. By influencing the immune system, the regulation of gut microbiota has the potential to enhance DKD renal inflammation, fibrotic damage, and proteinuria levels. In the treatment of DKD, targeted interventions utilizing herbal monomers and combinations, alongside interactions involving the host immune system and gut microbiota, play a crucial role.
